# Supplementary figures and images for: Early life stressful experiences escalate aggressive behavior in adulthood via changes in transthyretin expression and function
Source: eLife. 2022 Oct 13;11:e77968. doi: 10.7554/eLife.77968 (PMC9633068; doi:10.7554/eLife.77968)

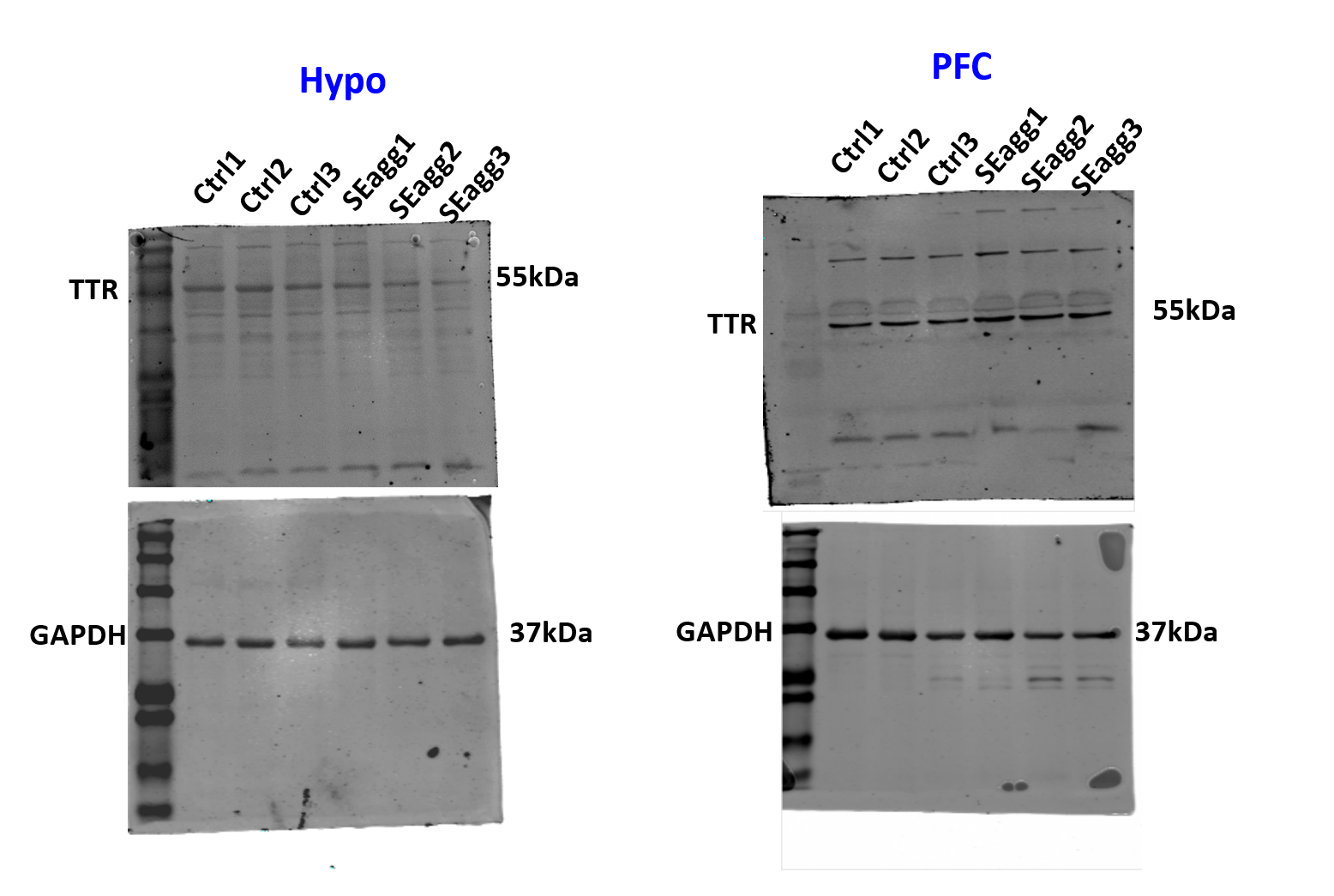

Supplement: Figure 3—source data 1. [file elife-77968-fig3-data1.zip › Figure 3-source data 1.tif]

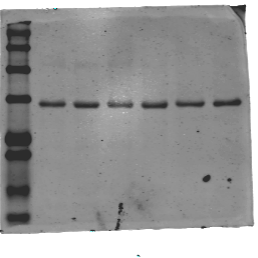

Supplement: Figure 3—source data 1. [file elife-77968-fig3-data1.zip › Hypo GAPDH.tif]

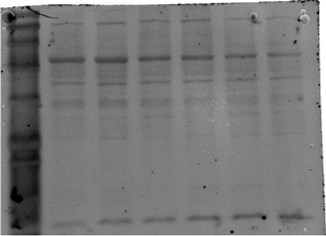

Supplement: Figure 3—source data 1. [file elife-77968-fig3-data1.zip › Hypo TTR.tif]

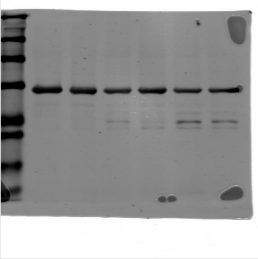

Supplement: Figure 3—source data 1. [file elife-77968-fig3-data1.zip › PFC GAPDH.tif]

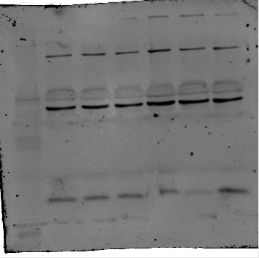

Supplement: Figure 3—source data 1. [file elife-77968-fig3-data1.zip › PFC TTR.tif]

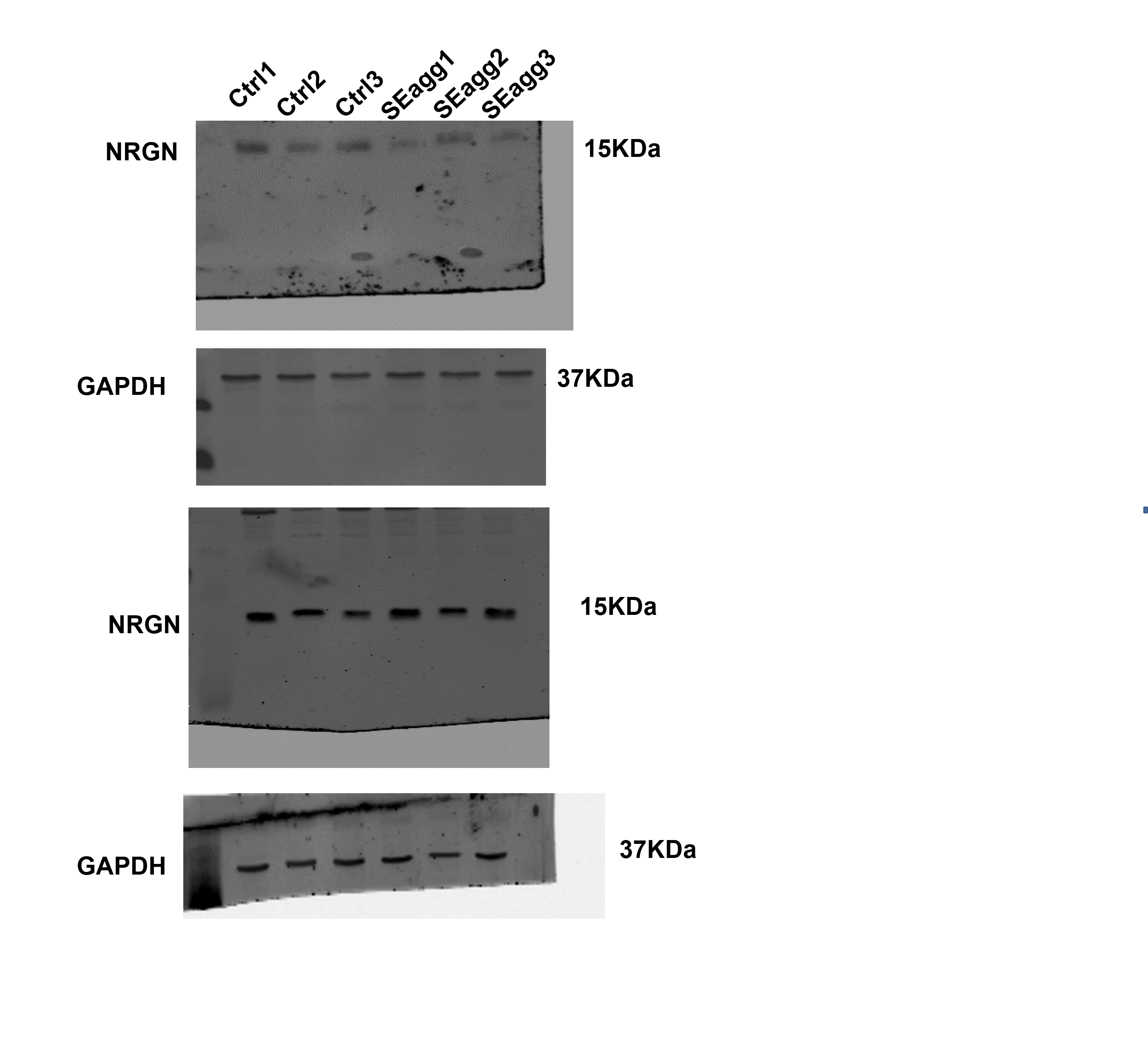

Supplement: Figure 4—source data 2. [file elife-77968-fig4-data2.zip › Fig 4- source data 2.tif]

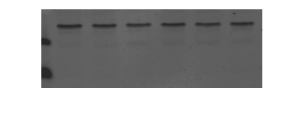

Supplement: Figure 4—source data 2. [file elife-77968-fig4-data2.zip › Hypo-GAPDH.tif]

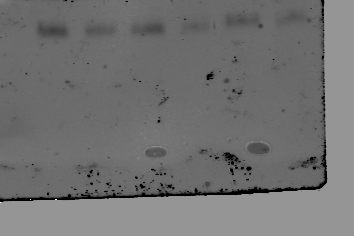

Supplement: Figure 4—source data 2. [file elife-77968-fig4-data2.zip › Hypo-NRGN.TIF]

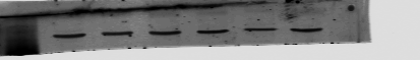

Supplement: Figure 4—source data 2. [file elife-77968-fig4-data2.zip › PFC-GAPDH.tif]

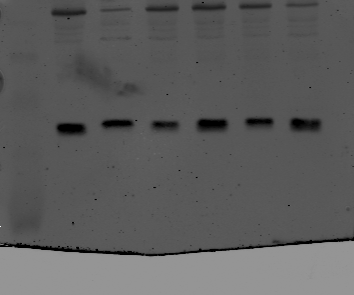

Supplement: Figure 4—source data 2. [file elife-77968-fig4-data2.zip › PFC-NRGN.TIF]
