# Supplementary material for: Early life stressful experiences escalate aggressive behavior in adulthood via changes in transthyretin expression and function
Source: eLife. 2022 Oct 13;11:e77968. doi: 10.7554/eLife.77968 (PMC9633068; doi:10.7554/eLife.77968)
Supplement: Transparent reporting form [file elife-77968-transrepform1.docx]

***eLife’s* transparent reporting form**

We encourage authors to provide detailed information *within their submission* to facilitate the interpretation and replication of experiments. Authors can upload supporting documentation to indicate the use of appropriate reporting guidelines for health-related research (see [EQUATOR Network](http://www.equator-network.org/%20)), life science research (see the [BioSharing Information Resource](https://biosharing.org/)), or the [ARRIVE guidelines](http://www.plosbiology.org/article/info:doi/10.1371/journal.pbio.1000412) for reporting work involving animal research. Where applicable, authors should refer to any relevant reporting standards documents in this form.

If you have any questions, please consult our Journal Policies and/or contact us: [editorial@elifesciences.org](mailto:editorial@elifesciences.org).

**Sample-size estimation**

- You should state whether an appropriate sample size was computed when the study was being designed
- You should state the statistical method of sample size computation and any required assumptions
- If no explicit power analysis was used, you should describe how you decided what sample (replicate) size (number) to use

Please outline where this information can be found within the submission (e.g., sections or figure legends), or explain why this information doesn’t apply to your submission:

Sample size was not predetermined during study design. However, for each experiment our sample sizes were based upon conventions established in literature including our own previously published papers that had used similar assays and power calculations. Sample size for each experiment is mentioned in the results and figure legends section.

**Replicates**

- You should report how often each experiment was performed
- You should include a definition of biological versus technical replication
- The data obtained should be provided and sufficient information should be provided to indicate the number of independent biological and/or technical replicates
- If you encountered any outliers, you should describe how these were handled
- Criteria for exclusion/inclusion of data should be clearly stated
- High-throughput sequence data should be uploaded before submission, with a private link for reviewers provided (these are available from both GEO and Array Express)

Please outline where this information can be found within the submission (e.g., sections or figure legends), or explain why this information doesn’t apply to your submission:

Information regarding number of experiments, total number of animals used in each experiment and animals used per group in each experiment have been provided in respective results and figure legend sections.

Each animal has been considered as a biological replicate and all our replicates represented in the figures are biological. In case of RT-PCR, Immunoblotting, ELISA and MedIP individual data points for each replicate has been plotted in the figures.

All the experiments except RNA sequencing (Figure 1 and 2) were repeated at least 2-3 times and with independent cohort of mice. RNA sequencing has been conducted from 3 mice per group from a single cohort.

For resident intruder behavioral test, phenotypic screening was performed based on different levels of aggression and accordingly animals were categorized into escalated aggressive, hyper-aggressive, moderate-aggressive, less aggressive and non-aggressive (Fig1). Screening parameters has been described in details in methods and results section.

Outliers, if found in were included in study. No data from experiments was excluded.

RNA sequencing data from Fig 1 and Fig 2 is accessible through GEO Series accession number GSE199844 and private link for reviewers have been provided. We have updated the GEO submission with FPKM values of all transcripts in each individual biological replicate.

ToreviewGEOaccessionGSE199844:
Goto <https://www.ncbi.nlm.nih.gov/geo/query/acc.cgi?acc=GSE199844>
Enter token afsxkauwnzizzkp into the box

**Statistical reporting**

- Statistical analysis methods should be described and justified
- Raw data should be presented in figures whenever informative to do so (typically when N per group is less than 10)
- For each experiment, you should identify the statistical tests used, exact values of N, definitions of center, methods of multiple test correction, and dispersion and precision measures (e.g., mean, median, SD, SEM, confidence intervals; and, for the major substantive results, a measure of effect size (e.g., Pearson's r, Cohen's d)
- Report exact p-values wherever possible alongside the summary statistics and 95% confidence intervals. These should be reported for all key questions and not only when the p-value is less than 0.05.

Please outline where this information can be found within the submission (e.g., sections or figure legends), or explain why this information doesn’t apply to your submission:

Statistical analysis method, sample sizes and p values for each experiment has been mentioned in the results and figure legend section. This has also been summarized in the statistical analysis section of the Materials and Methods. ANOVA summary tables has been added in main text and detailed table of respective figures has been uploaded as a single supplementary file.

Raw data has been presented in figures for experiments where N is less than 10 and also uploaded as source files. In addition raw data and source files for all behavioral experiments has been provided.

(For large datasets, or papers with a very large number of statistical tests, you may upload a single table file with tests, Ns, etc., with reference to sections in the manuscript.)

**Group allocation**

- Indicate how samples were allocated into experimental groups (in the case of clinical studies, please specify allocation to treatment method); if randomization was used, please also state if restricted randomization was applied
- Indicate if masking was used during group allocation, data collection and/or data analysis

Please outline where this information can be found within the submission (e.g., sections or figure legends), or explain why this information doesn’t apply to your submission:

At weaning, male and female mice were randomly assigned to control and peripubertal stress (PPS) conditions. Mice from different litters were mixed throughout the different home cages by placing equivalent numbers of animals from each litter into the control or experimental group and by avoiding having siblings in the same home cage. However, for evaluation for intergenerational inheritance experiment, litters originating from same parent i.e siblings were kept in same home cage. Information has been given in Materials and Methods section.

Resident intruder based scoring of aggressive behavior was performed by experimenter blinded to the animal identity and group assignment and has been mentioned in Materials and Methods section.

Experiments wherever possible were repeated by separate experimenters/researchers to ascertain reproducibility and independent verification of data and has been mentioned in Materials and Methods section.

**Additional data files (“source data”)**

- We encourage you to upload relevant additional data files, such as numerical data that are represented as a graph in a figure, or as a summary table
- Where provided, these should be in the most useful format, and they can be uploaded as “Source data” files linked to a main figure or table
- Include model definition files including the full list of parameters used
- Include code used for data analysis (e.g., R, MatLab)
- Avoid stating that data files are “available upon request”

Please indicate the figures or tables for which source data files have been provided:

Source data have been provided for the following main and supplementary figures

**Main Figures**-Fig.1C, Fig.3D, Fig. 4B-4I and Fig.4L, Fig.5B, 5C, Fig.6B-6D and Fig.7

**Supplementary Figures-** Fig. 4A-4D

Source data for RNA sequencing (Fig.1E and Fig.2B) have been uploaded to GEO for public accessibility (GEO accession GSE199844) and private link for reviewers have been provided.
